# A phylogenetic framework facilitates Y-STR variant discovery and classification via massively parallel sequencing

**DOI:** 10.1016/j.fsigen.2018.03.012

**Published:** 2018-07

**Authors:** Tunde I. Huszar, Mark A. Jobling, Jon H. Wetton

**Affiliations:** Department of Genetics & Genome Biology, University of Leicester, University Road, Leicester LE1 7RH, UK

**Keywords:** Y-STRs, Massively parallel sequencing, PowerSeq system, PPY23, Single nucleotide polymorphism (SNP), Repeat pattern variation (RPV)

## Abstract

Short tandem repeats on the male-specific region of the Y chromosome (Y-STRs) are permanently linked as haplotypes, and therefore Y-STR sequence diversity can be considered within the robust framework of a phylogeny of haplogroups defined by single nucleotide polymorphisms (SNPs). Here we use massively parallel sequencing (MPS) to analyse the 23 Y-STRs in Promega’s prototype PowerSeq™ Auto/Mito/Y System kit (containing the markers of the PowerPlex® Y23 [PPY23] System) in a set of 100 diverse Y chromosomes whose phylogenetic relationships are known from previous megabase-scale resequencing. Including allele duplications and alleles resulting from likely somatic mutation, we characterised 2311 alleles, demonstrating 99.83% concordance with capillary electrophoresis (CE) data on the same sample set. The set contains 267 distinct sequence-based alleles (an increase of 58% compared to the 169 detectable by CE), including 60 novel Y-STR variants phased with their flanking sequences which have not been reported previously to our knowledge. Variation includes 46 distinct alleles containing non-reference variants of SNPs/indels in both repeat and flanking regions, and 145 distinct alleles containing repeat pattern variants (RPV). For DYS385a,b, DYS481 and DYS390 we observed repeat count variation in short flanking segments previously considered invariable, and suggest new MPS-based structural designations based on these. We considered the observed variation in the context of the Y phylogeny: several specific haplogroup associations were observed for SNPs and indels, reflecting the low mutation rates of such variant types; however, RPVs showed less phylogenetic coherence and more recurrence, reflecting their relatively high mutation rates. In conclusion, our study reveals considerable additional diversity at the Y-STRs of the PPY23 set via MPS analysis, demonstrates high concordance with CE data, facilitates nomenclature standardisation, and places Y-STR sequence variants in their phylogenetic context.

## Highlights

• 23 Y-chromosomal STRs (PPY23) reanalysed by massively parallel sequencing.
• Phylogeny-based approach captures wide range of sequence variants in 100 samples.
• STR variants described in phase with their flanking sequences.
• Phylogenetic framework clarifies allele nomenclature and mutation processes.

## 1 Introduction

Classically, short tandem repeats (STRs) are divided into simple, compound, complex or even complex hypervariable types, reflecting the increasing complexity of the length, sequence and intermittent elements of building blocks [1]. However, conventional analysis of STR variation via capillary electrophoresis (CE) considers only overall length variation at such markers. Now that massively parallel sequencing (MPS) is being implemented in forensic typing, STRs are also becoming characterised by the richer range of variation displayed at the DNA sequence level, and this allows a more nuanced understanding of their diversity and the underlying mutation processes that generate this diversity.

One indication that increased allelic diversity is likely to be observed via MPS-based analysis of an STR is the complexity of the array [2], since repeat pattern variation (RPV) can arise from different numbers of repeat blocks with the same allele length (isometric alleles). Single nucleotide polymorphisms (SNPs) and insertions or deletions (indels) within repeat arrays can also contribute to diversity. While single nucleotide changes typically have very low mutation rates (∼10^−8^ per base per generation [3]) and therefore are unlikely to be observed as independent recurrences, the RPV in STRs mainly results from a more rapid (∼10^−3^ per repeat array per generation [4]) mutation process driven by replication slippage, so that the same variants can arise multiple times independently. SNPs and indels are not restricted to the repeat array, but are also found in the flanking regions, providing further basis for discrimination.

While autosomal STRs assort independently and are therefore uncorrelated, STRs on the male-specific region of the Y chromosome (MSY) are permanently linked together into a haplotype. This reduces the overall diversity that a Y-STR profile provides [5], but also means that Y-STR variation can be considered in the framework of a robust phylogeny of haplogroups defined by SNPs. Indeed, this relationship forms the basis of various methods that have been developed to predict MSY haplogroups from Y-STR haplotypes [[6], [7], [8], [9]]. Because of the high degree of population structure among Y chromosomes [10], studies of individual populations tend to capture a limited range of haplogroup diversity. Choosing samples for MPS-based Y-STR analysis to maximise haplogroup diversity, rather than on a population basis, should permit a broad survey of Y-STR sequence diversity to be undertaken efficiently. In addition, the phylogenetic framework should allow the degree of mutational recurrence of observed variants to be understood, with slow-mutating SNPs and indels tending to occur only once in the tree (monophyletic), and more rapidly-mutating RPVs showing recurrence (polyphyletic).

Here we select a set of 100 diverse samples in which MSY resequencing previously defined a highly resolved SNP-based phylogeny [11], and use MPS to sequence 23 Y-STRs in each. We describe the observed variants, suggest some improvements to MPS allele designations, and place the different classes of variants in their phylogenetic contexts.

## 2 Materials and methods

### 2.1 DNA samples

One hundred male DNA samples were selected from a previously described set of 448 [11]. Sample details are given in Table S1. Quantities of double-stranded DNA were verified prior to PCR using the Qubit® 2.0 fluorometer (Thermo Fisher Scientific) with the Qubit® dsDNA HS kit.

### 2.2 PCR amplification

Twenty-three Y-STRs (DYS19, DYS385a,b, DYS389I/II, DYS390, DYS391, DYS392, DYS393, DYS437, DYS438, DYS439, DYS448, DYS456, DYS458, DYS481, DYS533, DYS549, DYS570, DYS576, DYS635, DYS643 and Y-GATA-H4) were amplified from 0.5 ng template DNA using the prototype PowerSeq™ Auto/Mito/Y System (Promega) following the manufacturer’s recommended protocol. Results obtained for the autosomal STRs and the control region of mitochondrial DNA will be described elsewhere.

### 2.3 Library preparation and sequencing

Amplified products were purified using the MinElute® PCR purification kit (Qiagen), then quantified using the Qubit® dsDNA BR kit on the Qubit® 2.0 fluorometer.

Library preparation was performed on ∼500 ng product per sample using the TruSeq® DNA PCR-free LT (24-plex) and HT (96-plex) sample preparation reagents (Illumina). The manufacturer’s protocol was used, with an adjustment for the PowerSeq™ System (Promega), namely the use of the MinElute® PCR purification kit for size selection of amplicons.

Prepared libraries were quantified using the KAPA Library Quantification Kit for Illumina® platforms (KAPA Biosystems) with the LightCycler®480 (Roche) real-time PCR system following the manufacturers' recommendations. All indexed libraries were normalised to 4 nM, pooled at equal volumes and re-quantified using the same method to confirm pooled library concentration.

Pooled libraries were prepared for sequencing following the manufacturer’s protocol, diluting to 12 pM for loading and using a higher (15%) PhiX internal control library spike, as recommended for sequencing low-complexity libraries. Sequencing was performed on a MiSeq® FGx (Illumina) sequencer in ‘research use only’ (RUO) mode, via the “Generate FASTQ” workflow with “FASTQ Only” application and single-end (SE) method using MiSeq®v2 (300 cycles) reagent kits.

### 2.4 Data processing and analyses

Raw compressed fastq files were transferred from the MiSeq for external analysis. Quality checking was done by trimming any leftover of the known adapter sequences and low-quality read ends with Trimmomatic v0.32 [12] and SOAPec v2.01 [13] software. Resulting improvement in quality was confirmed using the FastQC v0.11.5 [14] programme.

The open-source software FDSTools v1.1.1 [15] was used to analyse reads spanning the STR repeat regions and their flanking regions.

Discovered variants were compared to the human genome reference sequence (GRCh38) and queried in dbSNP (build 151). Repeat pattern variants were compared to the existing literature (see *Results*) and the database STRBase ([16]; strbase.nist.gov, accessed 02-Nov-2017).

### 2.5 Relative read-depth ratio test for duplicated alleles

To distinguish between alleles resulting from somatic mutation and constitutive allele duplications, stutter-adjusted sequence read-depths for different PCR products were considered. This test is analogous to the semi-quantitative analysis of peak heights in CE, and assumes that similar size-range STRs in a multiplex reaction amplify and are detected comparably. When finding an additional allele (putative duplication) at a given STR in a sample, read depths of the same STR and a selected reference STR (another similar size-range marker amplified in the same reaction) were compared in the other analysed samples; this gave a range of expected relative read-depth ratios for those two STRs. The same comparison was then applied to each of the alleles of the putatively duplicated STR against the reference STR within the queried sample. This test indicated whether the two alleles were indeed duplicated (together displaying approximately double the expected read-depth ratio), or if the second allele is a likely result of somatic mutation (the summed ratios of both alleles lying in the expected range of a single-dose allele). Note that somatic mutants are only called when they do not lie in the −1 stutter position, to avoid confusion with stutter products.

## 3 Results

**Fig. 1:**
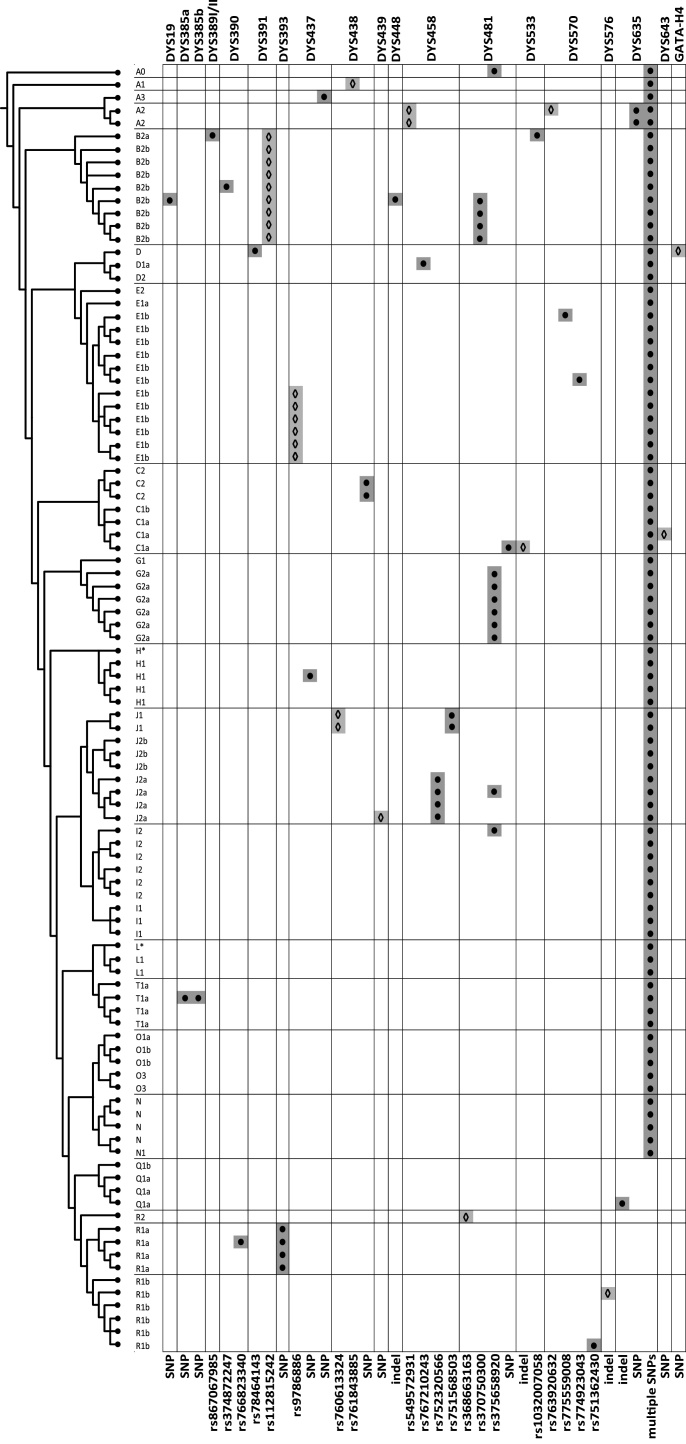
Observed SNPs and indels in their phylogenetic context. The phylogenetic tree to the left represents the relationships among 100 diverse Y chromosomes, based on 13,261 high-confidence Y-SNPs previously described [11]. Y-chromosome haplogroups are given in their shorthand formats (Table S1) to the right of the tree. Y-STR names are listed above. Variants are shaded in grey and represented by filled circles if internal to the repeat array, or unfilled diamonds if in the flanking region. Variants are described below, by rs# where available, or otherwise as ‘SNP’ or ‘indel’ (Table S3). Note that ‘multiple SNPs’ internal to DYS635 (which we regard as an RPV − see text) are found in 85/100 samples because the GRCh38 reference assembly carries the same derived state as superhaplogroup P, and hence all deeper-rooting clades bearing the ancestral state are considered as ‘alternative’ rather than ‘reference’ variants. Note that rs370750300 and rs375658920 are listed elsewhere as DYS481-associated SNPs, and thus included in the figure; however, we regard these as an RPV (see text).

In order to capture a wide range of Y-STR variants we took a phylogenetic approach, choosing a subset of one hundred DNA samples from a previously analysed set [11]. The published analysis had used massively parallel sequencing of ∼3.7 Mb of DNA in each of 448 diverse Y chromosomes, and constructed a maximum-parsimony tree based on a total of 13,261 SNPs. The subset here was selected to ensure that major clades and deep-rooting nodes of the tree were represented. The phylogenetic relationships of the analysed samples are represented schematically in Fig. 1, and with true branch lengths shown for comparison in Fig. S1. Details of samples, their MSY haplogroups and their populations of origin are given in Table S1. Samples were selected to establish a framework for maximum diversity, rather than to represent populations, and therefore classical population statistics are not applicable to our results.

We used Promega’s prototype PowerSeq™ kit to generate MPS data for 23 Y-STRs in the 100 samples. With the analytical threshold set to 20 × coverage, we observed a minimum-to-maximum per-allele sequence coverage of 251–11,600 × for 24-plex library preparation, and 72–11,906× for 96-plex library preparation. Per-sample, per-STR and per-run statistics are described in Table S2. We analysed a total of 2311 alleles in the 100 samples: as well as the expected 23 alleles per sample, this included eleven additional alleles, which we interpret as five allele duplications and six alleles arising via somatic mutation (Table S3), using a sequence read-depth approach to distinguish between the two classes (Fig. S2) (we also assume two alleles for each homoallelic combination of DYS385a,b).

### 3.1 Concordance of MPS data with CE-defined alleles

Sequence-derived repeat array lengths were compared to previously-determined CE-based PowerPlex® Y23 data [11]. Four of 2311 alleles (0.17%) were found to be discordant between the two methods (Table S4). Of these, one could be resolved by examining full-length sequence (an insertion of 13 bp in the flanking DNA), one by a SNP-based mobility shift that has been previously noted elsewhere [17], and the remaining two by possible differences in the positions of proprietary PCR primers for MPS and CE kits. Seven samples from diverse haplogroups were also analysed on the MiSeq FGx platform with the ForenSeq (Illumina/Verogen) kit (data not shown), showing full concordance of the 21 overlapping Y-STRs.

### 3.2 Diversity of observed alleles

Our samples contain a total of 267 distinct sequence-based Y-STR alleles, an overall 58% increase from the 169 length-based alleles distinguishable by CE (Table 1; Fig. S3). All but four Y-STRs showed increased allelic diversity when analysed by MPS. Observed isometric allele groups in the sample set are summarised in Table 2.

**Table 1:** Comparison of number of alleles for each Y-STR based on length only (as in CE) and on full sequence information (MPS).

Y-STR	Count of length-based alleles	Count of sequence-based alleles	Increase in number of alleles (%)	Novel sequence variants in this study
DYS389II	7	32	357.1	4
DYS390	8	19	137.5	6
DYS448	9	19	111.1	5
DYS391	5	10	100.0	6
DYS437	5	9	80.0	2
DYS481	12	21	75.0	3
DYS458	10	16	60.0	9
DYS385a,b	14	22	57.1	7
DYS635	11	17	54.5	4
DYS570	8	12	50.0	4
DYS438	7	10	42.9	2
DYS389I	5	6	20.0	0
DYS439	5	6	20.0	1
DYS19	6	7	16.7	1
DYS393	6	7	16.7	0
Y-GATA-H4	6	7	16.7	1
DYS533	8	9	12.5	2
DYS643	9	10	11.1	2
DYS392	8	8	0.0	0
DYS456	5	5	0.0	0
DYS549	6	6	0.0	0
DYS576	9	9	0.0	1
Total	169	267	58.0	60

Abbreviations: Y-STR, Y-chromosomal short tandem repeat; CE, capillary electrophoresis; MPS, massively parallel sequencing.

STRs are listed in descending order of percentage increase in number of alleles, based on sequence-level information from MPS.

**Table 2:** Sum of isometric allele groups of 23 Y-STRs analysed by MPS found in the sample set.

	# of MPS alleles found per single CE allele							
	2	3	4	5	6	7	8	9
total # of isometric allele groups	41	13	2	1	–	1	1	1
# of Y-STRs with isometric allele groups	19	10	2	1	–	1	1	1

Abbreviations: Y-STR, Y-chromosomal short tandem repeat; MPS, massively parallel sequencing; CE, capillary electrophoresis.

Isometric allele groups are alleles with the same fragment length, but showing different sequences.

Although an online resource to collect STR sequence variation under an international collaboration is due to be hosted by NCBI as STRSeq BioProject [18], data for the Y-Chromosomal STR loci sub-project (Accession: PRJNA380347) are not yet available for query (https://www.ncbi.nlm.nih.gov/bioproject/380347l, last accessed 03-Jan-2018). We therefore compared our results to the current published literature [16,17,[19], [20], [21], [22], [23], [24], [25], [26], [27], [28], [29]] strbase.nist.gov, accessed 02-Nov-2017 (details in Table S5), and in Table 3 we describe 60 novel Y-STR variants in phase with their flanking sequences not reported elsewhere, to our knowledge.

**Table 3:** List of novel Y-STR sequence variants defined by MPS.

Y-STR	Y-STR definition; Novel sequence variants	Observed #		Aspects of novelty
**DYS19**	*[TCTA]a ccta [TCTA]b* [ref. 30]			
	CE12_TCTA[13]_a+b_ ccta[0]	1		SNP internal to repeat array, allele name is a + b − 1 for compatibility to CE

**DYS385a,b**	*DYS385a [TTTC]a/DYS385b [GAAA]a* [ref. 30] *DYS385a,b [aagg]5-9 [GAAA]a* (this study)			
	CE9_AAGG[5]GAAA[10]	1		new combination of repeat units; upstream flanking region previously considered non-variable, but shows high level of variation in number of repeats; therefore here considered part of the repeat array as AAGG[5–9] [23] also found AAGG[9])
	CE13_AAGG[5]GAAA[14]	1		
	CE15_AAGG[5]GAAA[16]	1		
	CE15_AAGG[8]GAAA[13]	1		
	CE16_AAGG[8]GAAA[14]	1		
	CE17_AAGG[5]GAAA[18]	1		
	CE18_AAGG[7]GAAA[17]	2		

**DYS389II**	*[TAGA]a [CAGA]b N48 [TAGA]c [CAGA]d* [ref. 30]			
	CE30_TAGA[11]CAGA[2]N[48]TAGA[13]CAGA[4]	1		shorter first CAGA array
	CE30_TAGA[9]CAGA[3]N[48]TAGA[12]CAGA[6]	2		new combination of repeat units
	CE31_TAGA[10]CAGA[3]N[48]TAGA[11]CAGA[1]TAGA[1]CAGA[5]	1		SNP internal to repeat array
	CE34_TAGA[10]CAGA[3]N[48]TAGA[15]CAGA[6]	1		longer second TAGA array

**DYS390**	*[TAGA]a [CAGA]b [TAGA]c [CAGA]d* [ref. 30] *[TAGA]a [CAGA]b [TAGA]c [CAGA]d [taga]1-3* (this study)			
	CE22_TAGA[14]_a+c_CAGA[0]CAGA[8]TAGA[2]	1		SNP internal to repeat array
	CE23_TAGA[5]CAGA[1]TAGA[9]CAGA[8]TAGA[2]	1		longer first TAGA array
	CE24_TAGA[4]CAGA[1]TAGA[10]CAGA[10]TAGA[1]	1		longer second CAGA/shorter third TAGA array
	CE24_TAGA[4]CAGA[1]TAGA[11]CAGA[7]TAGA[3]	1		longer third TAGA array
	CE24_TAGA[4]CAGA[1]TAGA[11]CAGA[8]TAGA[1]GAGA[1]	1		SNP internal to repeat array
	CE26_TAGA[4]CAGA[1]TAGA[12]CAGA[9]TAGA[2]	2		new combination of repeat units

**DYS391**	*[TCTA]a* [ref. 30]			
	CE8_TCTA[8]_+50C>A rs112815242 @11,982,182 M8738/CTS1866	2		SNP in the flanking region
	CE9_TCTA[9]_+50C>A rs112815242 @11,982,182 M8738/CTS1866	2		SNP in the flanking region
	CE10_TCTA[10]_+50C>A rs112815242 @11,982,182 M8738/CTS1866	2		SNP in the flanking region
	CE11_TCTA[11]_+50C>A rs112815242 @11,982,182 M8738/CTS1866	2		SNP in the flanking region
	CE11_TCTG[1]TCTA[10]	1		SNP internal to repeat array
	CE12_TCTA[12]_+50C>A rs112815242 @11,982,182 M8738/CTS1866	1		SNP in the flanking region

**DYS437**	*[TCTA]a [TCTG]b [TCTA]4* (STRBase, accessed on 03 Nov 2017)			
	CE15_TCTG[1]TCTA[8]TCTG[2]TCTA[4]	1		SNP internal to repeat array
	CE16_TCTA[6]TCTG[1]TCTA[3]TCTG[2]TCTA[4]	1		SNP internal to repeat array

**DYS438**	*[TTTTC]a* [ref. 30]			
	CE8_TTTTC[8]_+21T>C rs761843885 @12,825,969 Z10613	1		shorter array; SNP in the flanking region
	CE11_TTTTC[11]_+7A>C rs760613324 @12,825,955 L255/PF4706	1		SNP in the flanking region

**DYS439**	*[GATA]a* [ref. 30]			
	CE11_GATA[11]_+3A>T SNP @12,403,567	1		SNP in the flanking region

**DYS448**	*[AGAGAT]a N42 [AGAGAT]b* [ref. 30]			
	CE13_AGAGAT[5]N[42]AGAGAT[8]	1		shorter first AGAGAT array
	CE19_AGAGAT[13]N[42]AGAGAT[6]	1		shorter second AGAGAT array
	CE20.4_AGAGAT[3]AGAT[1]AGAGAT[9]N[42]AGAGAT[8]	1		indel in the repeat array
	CE23_AGAGAT[14]N[42]AGAGAT[9]	1		new combination of repeat units
	CE23_AGAGAT[15]N[42]AGAGAT[8]	1		longer first AGAGAT array

**DYS458**	*[GAAA]a* [ref. 28]			
	CE14_GAAA[13]GGAA[1]	1		SNP internal to repeat array
	CE15_GAAA[14]GGAA[1]	1		SNP internal to repeat array
	CE16_GAAA[15]GGAA[1]	1		SNP internal to repeat array
	CE17_GAAA[17]_+32T>C rs549572931 @7,999,934 M11097	1		SNP in the flanking region
	CE17.2_GAAA[15]AA[1]GAAA[2]	1		indel in the repeat array
	CE19_GAAA[19]_+32T>C rs549572931 @7,999,934 M11097	1		SNP in the flanking region
	CE19_GAAG[1]GAAA[18]	1		SNP internal to repeat array
	CE19.2_GAAA[17]AA[1]GAAA[2]	1		indel in the repeat array
	CE20_GAAA[19]GGAA[1]	1		SNP internal to repeat array

**DYS481**	*[CTT]a* [ref. 30] *[ctg]0-2 [CTT]a* (this study)			
	CE26_CTG[0]CTT[27]	1		new combination of repeat units
	CE27_CTG[0]CTT[28]	2		new combination of repeat units
	CE28_CTG[1]CTT[3]CCT[1]CTT[24]	1		SNP internal to repeat array

**DYS533**	*[TATC]a* [ref. 30]			
	CE14.1_TATC[11]_−48.1->CTCTTCTAACTAT indel @16,281,301	1		indel in the flanking region
	CE15_TATC[15]	1		longer repeat unit in array

**DYS570**	*[TTTC]a* [ref. 30]			
	CE16_TTTC[16]_+4T>G rs763920632 @6,993,261 PH250	1		SNP in the flanking region
	CE17_TTCC[1]TTTC[16]	1		SNP internal to repeat array
	CE17_TTTC[15]CTTC[1]TTTC[1]	1		SNP internal to repeat array
	CE19_TTTC[5]TCTC[1]TTTC[13]	1		SNP internal to repeat array

**DYS576**	*[AAAG]a* [ref. 30]			
	CE17.1_AAAG[18]_+3AAA>− indel @7,185,388	1		indel in the flanking region

**DYS635**	*[TAGA]a [TACA]b [TAGA]c [TACA]d [TAGA]e [TACA]f [TAGA]g* [ref. 30]			
	CE18_TAGA[8]TACA[2]TAGA[2]TACA[2]TAGA[4]	3		new combination of repeat units
	CE20_TAGA[8]CAGA[1]TAGA[1]TACA[2]TAGA[2]TACA[2]TAGA[4]	1		SNP internal to repeat array
	CE21_TAGA[9]CAGA[1]TAGA[1]TACA[2]TAGA[2]TACA[2]TAGA[4]	1		SNP internal to repeat array
	CE25_TAGA[14]TACA[3]TAGA[2]TACA[2]TAGA[4]	1		SNP internal to repeat array

**DYS643**	*[CTTTT]a* [ref. 30]			
	CE11_CTTTT[11]_−7A>G SNP @15,314,125	1		SNP in the flanking region
	CE15_CTTTT[15]	1		longer repeat unit in array

**Y-GATA-H4**	*[TCTA]a* [ref. 30]			
	CE13_TCTA[13]_+36A>G SNP @16,631,756 Y15322/Z34275	1		SNP in the flanking region

Abbreviations: Y-STR, Y-chromosomal short tandem repeat; MPS, massively parallel sequencing; SNP, single nucleotide polymorphism; CE, capillary electrophoresis.

For DYS19, DYS385a,b, DYS390 and DYS481, uncounted repeat units are denoted with lower-case letters within the Y-STR definition. GRCh38 chrY genomic positions are noted after the ‘@’ signs. rs# or names of SNPs/indels are provided where available.

These sequence variants, in phase with their flanking sequences, to the best of our knowledge, have not been described in the literature previously [16,17,[19], [20], [21], [22], [23], [24], [25], [26], [27], [28], [29]], strbase.nist.gov, accessed 02-Nov-2017. Comparison is detailed in Table S5.

Newly arising Y-STR variants may result from single-nucleotide changes (SNPs) or insertions or deletions (indels) affecting the repeats themselves, or the flanking regions. We have found 22 different SNPs or indels in the repeat regions in 27 distinct alleles of 15 Y-STRs, in 27 of the 100 samples. It is of paramount importance to analyse full-length sequences, rather than solely the repeat region, because the flanking regions contribute to the analysed length, and their omission can therefore lead to discordance with CE-based allele calls (as seen, for example, for DYS533 in Table S4). We therefore also describe 12 different flanking region SNPs or indels in 19 distinct alleles of 11 Y-STRs; such flanking-region variants are observed in 26 of the 100 analysed samples. Altogether we describe 34 different SNPs or indels in 46 distinct alleles of 19 Y-STRs, observed in 43 of the 100 samples.

The other class of variants is defined by repeat pattern variation (RPV), in which arrays with more than one block of repeats present different combinations of units adding up to the same overall length, and therefore indistinguishable by CE (isometric alleles). We describe 145 distinct alleles showing RPV affecting nine Y-STRs; such alleles are observed in all analysed samples.

While Y-STRs, with the exception of DYS385a,b, are expected to present only one allele, in our sample set we observed several examples showing more than one (which could be either duplications or somatic mutations; Table S3), one of which was only detected by MPS. In a haplogroup C1a sample, two isometric alleles of DYS643 were detected (Table S3), and distinguished by a flanking A to G SNP upstream of the 11 CTTTT repeats in one allele, but not in the other.

To represent the observed sequence-level variation in a visually comprehensible way, we used Microsoft Excel to build a compressed and uniform summary of the allele range and internal structure of each of the Y-STRs (Table S6). All variants with indels or SNPs, either internal to the arrays or in the flanking regions, are summarised in Tables S7–S8. All variants for each allele and sample are listed in a bracketed format in Table S9, and complete sequence strings for alleles are listed in Table S10.

### 3.3 Novel variants with implications for nomenclature

This study focused on capturing a wide range of sequence variants through MPS analysis of Y-STRs, rather than taking a population-based approach [19,20,23,24]. The consequent observation of rare variants suggests a broader framework of sequence-level variation that is not always obvious in population studies. Considering rare variants within this framework leads us to suggest improvements in the MPS-based reporting of alleles for three Y-STRs − DYS385a,b and DYS481 (both previously considered simple repeats), and DYS390.

For DYS385a,b, nomenclature is complicated by the fact that the two copies of the STR lie on opposite strands, and the ISFG recommendation [30] is to report sequences based only on the forward-strand direction, leading to different repeat designations for the a and b copies. However, current commercial kits do not distinguish between the two forms, so in order to minimise confusion, we choose to follow a description based on the b copy (forward strand), because the GRCh38 human genome reference sequence for DYS385b is AAGG[6]GAAA[14], consistent with the classical, pre-MPS era repeat designation of GAAA[n]. However, while the majority of our samples indeed carry alleles containing six AAGG flanking repeats, we also observe examples showing variation in this block (Table 4). This, together with variants observed by others [23], leads us to suggest a structure described as AAGG[5-9]GAAA[n].

**Table 4:** Summary of MPS sequence variants showing sequences previously considered as non-variable flanking regions.

Y-STR	Allele	Observed #	General structure of alleles including variable flanking sequences	CE allele name designation	Examples in this study	
DYS385a,b	canonical	193	**AAGG[6]**GAAA[n]	n	CEU-NA12716	CE11_**AAGG[6]**GAAA[11], CE14_**AAGG[6]**GAAA[14]
	variant	4	**AAGG[5]**GAAA[n]	n − 1	kun-m82	CE15_**AAGG[5]**GAAA[16], CE17_**AAGG[5]**GAAA[18]
	variant	2	**AAGG[7]**GAAA[n]	n + 1	TSI-NA20805	CE10_**AAGG[6]**GAAA[10], CE18_**AAGG[7]**GAAA[17]
	variant	2	**AAGG[8]**GAAA[n]	n + 2	bkl-46	CE15_**AAGG[8]**GAAA[13], CE16_**AAGG[8]**GAAA[14]
	variant		**AAGG[9]**GAAA[n]	n + 3	in [23]	

DYS481	canonical	87	**CTG[1]**CTT[n]	n	CEU-NA12716	CE23_**CTG[1]**CTT[23]
	variant	9	**CTG[2]**CTT[n]	n + 1	tur-1	CE21_**CTG[2]**CTT[20]
	variant	4	**CTG[0]**CTT[n]	n − 1	bak-55	CE27_**CTG[0]**CTT[28]

DYS390	canonical	97	TAGA[n]CAGA[o]TAGA[p]CAGA[q]**TAGA[2]**	(n + o + p + q)	CEU-NA12716	CE24_TAGA[4]CAGA[1]TAGA[11]CAGA[8]**TAGA[2]**
	variant	2	TAGA[n]CAGA[o]TAGA[p]CAGA[q]**TAGA[1]**	(n + o + p + q) − 1	bhu-1150	CE24_TAGA[4]CAGA[1]TAGA[10]CAGA[10]**TAGA[1]**
	variant	1	TAGA[n]CAGA[o]TAGA[p]CAGA[q]**TAGA[3]**	(n + o + p + q) + 1	bav-55	CE24_TAGA[4]CAGA[1]TAGA[11]CAGA[7]**TAGA[3]**

Abbreviations: MPS, massively parallel sequencing; Y-STR, Y-chromosomal short tandem repeat; CE, capillary electrophoresis.

The most frequent allele variants are denoted ‘canonical’; repeat units that show additional polymorphism are shown in bold.

For DYS481, the GRCh38 reference assembly contains an array of 22 CTT repeats, preceded by the trinucleotide CTG. However, we observe sequence-based alleles lacking this CTG, and also alleles containing two CTG copies (Table 4). Similar variants have been reported before [[22], [23], [24]], but were described in terms of SNP variants. We suggest applying the same principle as above, and reporting sequence variants at DYS481 as CTG[0-2]CTT[n].

DYS390 is already considered to be a compound Y-STR [27] and in the GRCh38 reference assembly is represented as TAGA[4]CAGA[1]TAGA[11]CAGA[8] followed by a TAGATAGA flanking sequence that is considered non-variable. We find that most of our samples carry alleles similar to the reference in the latter respect; however, we also observe the flanking sequence to exist as a variable number of TAGA repeats, TAGA[1-3] (Table 4). DYS390 sequence variants would thus be described as TAGA[n]CAGA[o]TAGA[p]CAGA[q]TAGA[1-3].

In summary, therefore, we suggest that these units are added to the MPS-based reporting of DYS385a,b, DYS481 and DYS390 alleles for clarity, but remain uncounted in CE allele names for compatibility with existing nomenclature.

### 3.4 Phylogenetic association of variants

Based on our sequence data, Y-STRs can be classified into two groups. Certain simple (DYS391, DYS392, DYS393, DYS438, DYS439, DYS456, DYS458, DYS533, DYS549, DYS570, DYS576 and DYS643) and compound (DYS19 and Y-GATA-H4) STRs contain only one variable-length array of repeats, which is the source of the overall length variation. In these STRs, sequence variants result from SNPs and indels either within the array or in the flanking regions (Table S7). By contrast, DYS385a,b, DYS389I, DYS389II, DYS390, DYS437, DYS448, DYS481 and DYS635 all contain combinations of more than one variable-length array of repeats, which combine to generate the overall length variation (Table S11). Sequence variants can therefore result not only from SNPs and indels, but also from RPV in which isometric alleles differ in the numbers of each repeat component.

Different variant types have different underlying mutation processes and rates. While SNPs and small indels have low mutation rates (for SNPs, ∼10^−8^ per generation [31], and slower for indels [32]), the replication-slippage-based mechanisms that affect STR repeat arrays have much higher rates: these are length-dependent, but are typically five orders of magnitude greater than those of SNPs [33,34]. We therefore expect variant alleles involving SNPs and indels to show clearer phylogenetic coherence than those involving RPVs.

#### 3.4.1 Phylogenetic association of SNPs/indels

Previous studies have described a number of Y-STR sequence variants that are associated with particular haplogroups, and some of these associations are also confirmed here (Fig. 1; Table S7). One example is the shortening of a CAGA repeat block within DYS390 [27] (corresponding to block q in the notation given above, and also known as the DYS390.1 deletion), previously reported to be associated with a sub-haplogroup of C [35]. A second example is an indel within the DYS458 repeat array, generating intermediate (.2) alleles, and associated with haplogroup J1 [36].

The additional SNPs and indels we observe also include several novel haplogroup associations, and a low degree of recurrent mutation, as expected (Fig. 1; Table S7). Examples include a DYS391 flanking SNP (rs112815242) seen in all nine haplogroup B2 samples in our study, and the presence of a DYS393 internal SNP (A to C at the first base of the AGAT[n] repeat array) in all four haplogroup R1a samples (Fig. 1; Table S7): this was also seen in a haplogroup R1a individual analysed in a previous study [24].

#### 3.4.2 Phylogenetic association of RPVs

**Fig. 2:**
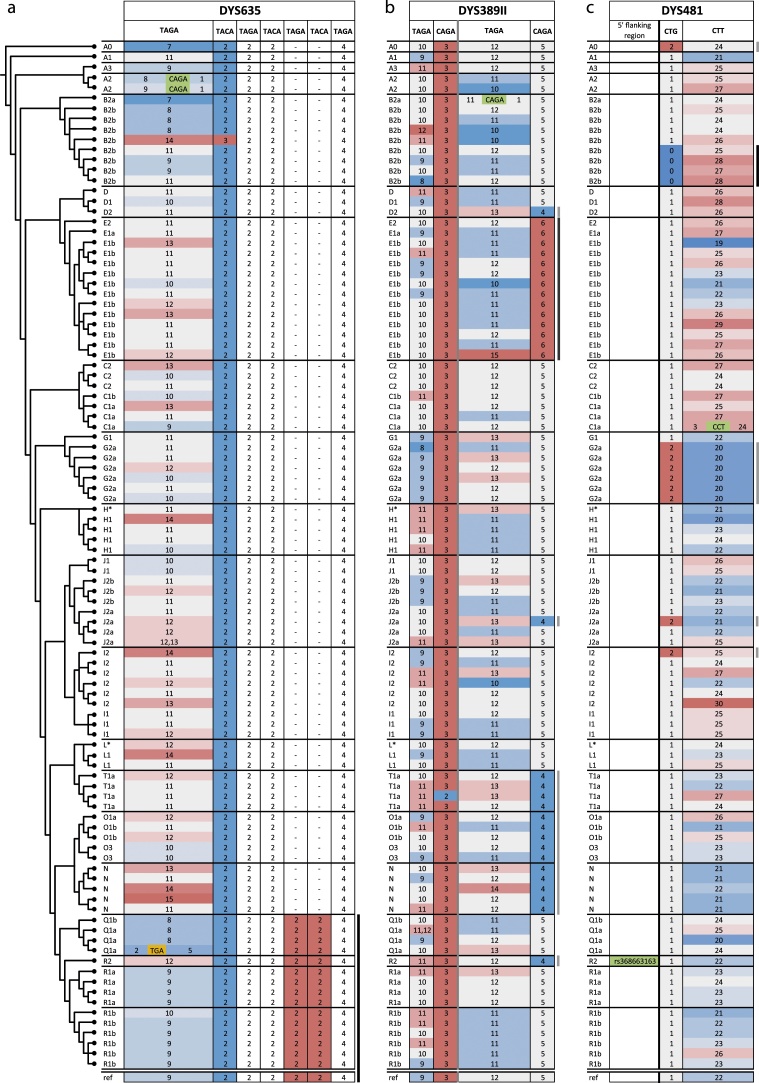
Examples of observed RPVs in their phylogenetic contexts. A phylogenetic tree is shown to the left, as in Fig. 1. a) Allele structures for DYS635 in all 100 samples. Repeat unit sequences are shown above, and boxes below contain the number of repeat units in each block, coloured by heat-map from blue (shortest) to red (longest). Invariant blocks are not coloured. SNPs and indels are highlighted by green and orange boxes respectively. Bars on the right mark features specifically mentioned in the text, and are coloured black for monophyletic, or grey for polyphyletic examples. Below is represented the reference sequence allele structure (‘ref.’) in GRCh38 chrY. To fully appreciate the colours of the heat-map, please, consult the online version of the figure. b) Allele structures for DYS389II; c) Allele structures for DYS481.

Despite the relatively high mutation rates of Y-STRs, allele lengths are well-known to be non-randomly associated with the phylogeny, and we observe this in our data (Fig. S4). Similarly, some associations between RPVs and particular haplogroups are detectable here. One clear example is seen in the exclusive association of an RPV in the compound STR DYS635 with the fifteen superhaplogroup P (containing Q, R) samples (Fig. 2a): this variant, which features two additional repeat blocks compared to more ancestral haplogroups, is unlikely to arise independently multiple times. A haplogroup Q1a sample with a DYS635 21.3 allele carrying an internal indel on the background of this RPV (see Fig. 2a) allows the observation of these two types of variants relative to each other, and indicates that the RPV occurred prior to this indel. Previous sequencing of intermediate .3 alleles [37] has not revealed any other underlying structure for these variants apart from that described here, therefore Y chromosomes with such CE alleles are most likely to belong to the same phylogenetic lineage as our Q1a case.

DYS389II provides a second example, where one short repeat block has a narrow range of variation (CAGA[4-6]), and hence a probable low mutation rate; in our sample of 100 chromosomes, 6-repeat blocks appear monophyletic, being seen only in the fourteen haplogroup E samples, while 4-repeat blocks are polyphyletic, observed in all fourteen haplogroup T, O and N samples, but also appearing sporadically elsewhere in the phylogeny (Fig. 2b).

A third example, DYS481, shows a monophyletic RPV (absence of the initial CTG repeat; see *Novel variants with implications for nomenclature* section above) in a sub-clade of haplogroup B2b (Fig. 2c); by contrast, presence of two copies of this CTG repeat is polyphyletic, though its combination with CTT[20] is confined to haplogroup G2a in our samples.

## 4 Discussion

Here, we have described DNA sequence variation in the 23 Y-STRs of the prototype PowerSeq™ Auto/Mito/Y System within a set of 100 diverse Y chromosomes whose phylogenetic relationships have been previously determined via megabase-scale resequencing [11]. Of the 2311 STR alleles observed in our dataset, 267 are distinguishable by MPS analysis, compared to just 169 based on length-discrimination via CE (Table 1). Use of a phylogenetic framework enhances the observed STR sequence diversity compared to a typical population study (Table S5), and allows us to consider how variants arise via different mutation processes with different rates. It also provides a wider perspective to recognise additional variable sequences adjacent to classical arrays. The inclusion of these features in the reporting of sequence-based alleles should facilitate more harmonious nomenclature across different workflows and platforms.

One limitation of our study is its small overall sample size. This means that, while some haplogroups are represented multiple times and therefore provide evidence for coherent associations with particular Y-STR sequence variants, others are singletons, and therefore the status of observed variants is unclear (Fig. 1, Table S7). In principle, these could also be true singletons, or they could be shared among a set of unobserved phylogenetically related Y chromosomes. Studies of larger sets of well-characterised Y chromosomes should address this.

As in other recent MPS-based studies of forensically-relevant STRs [2,23], we observed a positive relationship between STR complexity and the number of sequence variants captured. Most of the newly-described variants in our study originate from complex underlying structures (RPVs), while variants arising from SNPs and indels are independent of structure, and affect almost all the Y-STRs studied, regardless of complexity. These two main types of variants (RPVs, and SNPs/indels) were expected to present different patterns within the phylogeny due to their different likely mutation rates. This expectation was indeed realised (Fig. 1, Fig. 2), with RPVs rarely corresponding to a single event, but several monophyletic occurrences being observed for SNPs or indels.

STR sequencing demonstrates the importance of flanking region variation: omitting the reporting of indels from these areas may result in CE/MPS discordance and could jeopardise the back-compatibility of allele calls. While differences in primer design may result in discordances due to inclusion/exclusion of indels (see Table S4), another less obvious issue came to light in our dataset, namely a fragment mobility shift arising from flanking SNP variation (see Table S4). This phenomenon has been described for other STRs [38,39], but only recently for DYS481 [17]. Here, we observed the same flanking SNP as described previously [17], resulting in the same discordance between sequence length and CE results (see Table S4). This SNP (rs368663163, also known as L266 and PF6108) is phylogenetically associated with haplogroup R2 in the ISOGG tree (Y-DNA Haplogroup Tree 2017, Version: 12.320), and occurs in the single haplogroup R2 sample in our study. The mobility shift was noticed inconsistently in previous studies, due to different DYS481 primer designs: in some designs (and in the Yfiler® Plus kit), a primer bridges the SNP, thus masking its CE mobility shift effect [29,34,[40], [41], [42], [43], [44], [45]], while in others (and in the PowerPlex® Y23 and PowerSeq™ Y kits) the primers encompass the SNP, leading to a DYS481 .1 allele [17,46]. One study [47] found 20 among 270 Pakistani males to carry DYS481 .1 alleles, and used SNP typing to assign them all to haplogroup R2-M479. This haplogroup association can be further supported by surveying a large global PPY23 dataset [48], in which all 26 samples carrying DYS481 .1 alleles are predicted to belong to haplogroup R2 using the NevGen predictor, a tool whose accuracy has been recently assessed [49]. These observations support our singleton finding, and suggest rs368663163 as a strong indicator of haplogroup R2, and of the geographical regions (South and Central Asia [50,51]) in which this lineage is prevalent.

Currently the most notable general effect of applying MPS to forensic STRs is the resulting increase in allele diversity, largely originating from RPVs, and the resolution by sequence variants of a proportion of length-homozygous alleles as isometric heterozygotes. We have shown here that MPS-based analysis of STRs on the Y chromosome also increases allele diversity, and hence haplotype diversity, and that it has potential to distinguish between isometric alleles of bilocal Y-STRs. Much effort has been devoted to elevating the discriminatory power of Y-STR typing by increasing the number of STRs analysed [40], and by focusing on sub-sets that have particularly high mutation rates (rapidly mutating STRs; RM Y-STRs [52,53]). Applying MPS to additional STRs, including RM Y-STRs, is expected to increase discriminatory power as allele diversity increases. However, as our phylogenetically-based data show, within a patrilineage, additional variation from SNPs and indels is unlikely to be observed because of the associated low mutation rates of these events. Any additional variation at this scale will come from RPVs which, while mutating more rapidly than SNPs and indels, appear to have mutation rates that are lower than the rate of overall STR length variation. If this is so, individual male identification via MSY analysis may not be greatly advanced by applying MPS approaches. However, the association between SNPs and STRs is likely to be beneficial for the analysis of multi-male mixtures via MPS. If SNPs/indels prove to be phylogenetically restricted, as we observe, they will be associated with the characteristic Y-STR allele lengths, which have previously been exploited for haplogroup prediction [[6], [7], [8], [9]]. Knowledge of the apparent mixture ratio of the contributing haplogroups from SNP/indel variants may help with the deconvolution of mixtures when the two haplogroups have very distinct allele size ranges at particular loci. Furthermore, our current data on isometric alleles suggest that insights will also be provided into relative stutter ratios between pure and interrupted repeat array structures.

## Conflicts of interest

None.
