# Supplementary material for: A phylogenetic framework facilitates Y-STR variant discovery and classification via massively parallel sequencing
Source: Forensic Sci Int Genet. 2018 Jul;35:97–106. doi: 10.1016/j.fsigen.2018.03.012 (PMC6010625; doi:10.1016/j.fsigen.2018.03.012)
Supplement: Supplementary file 1 [file mmc1.pdf]

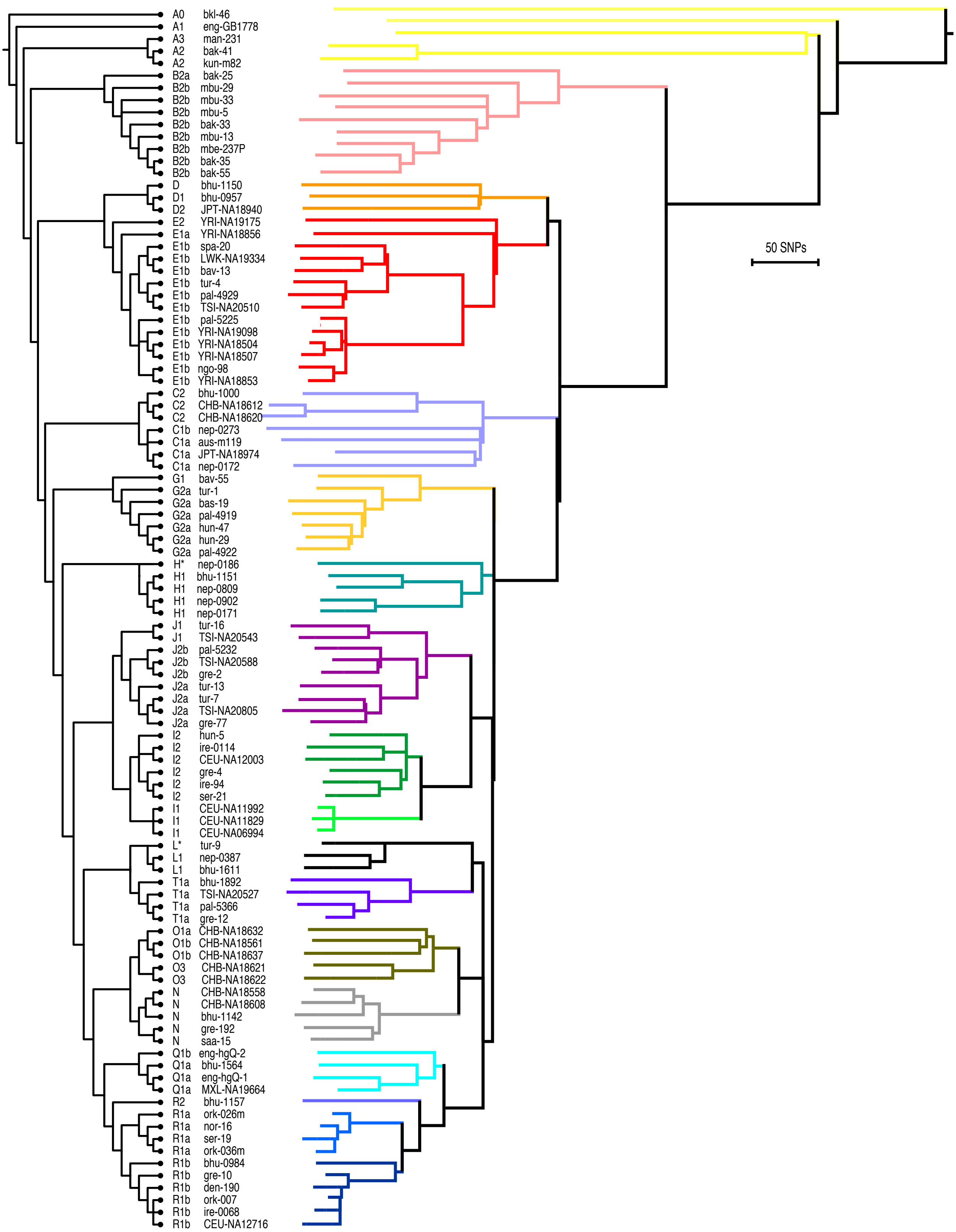

Figure S1: MSY phylogeny showing true branch lengths.

To the left is the schematic phylogeny relating the MSY haplogroups of the100 samples in this study, as used in Figures 1 and 2, with haplogroup and sample names (as in Table S1) given at the tips. To the right is the same phylogeny, but with branch lengths based on the number of SNPs discovered in resequencing of 3.7 Mb of MSY DNA in each sample (Hallast et al., Mol. Biol. Evol. 32 [2015] 661-73). A scale bar representing 50 SNPs is shown.

a

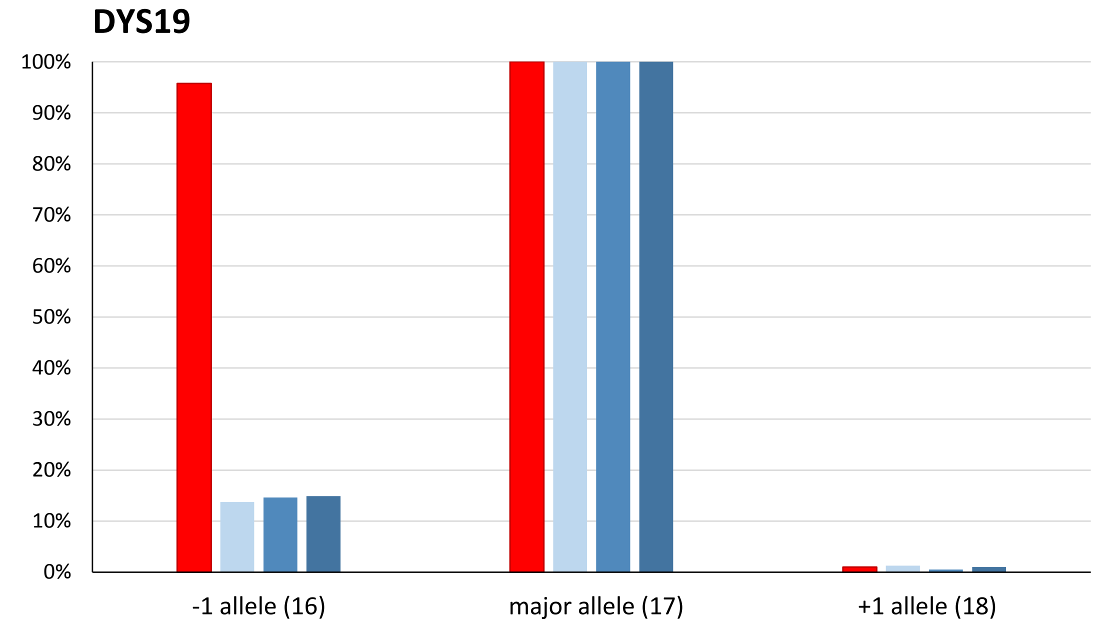

	-1 allele (16)	major allele (17)	+1 allele (18)
MEAN%	14.42%	100%	0.93%
STDEV%	0.49%	0%	0.32%
bhu-1000	95.75%	100%	1.02%

b

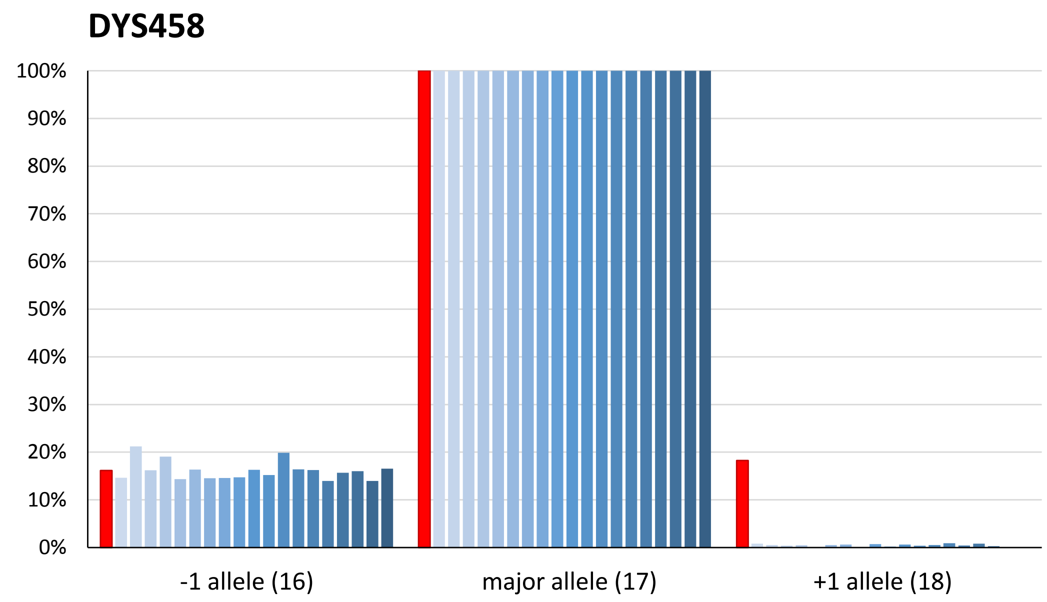

	-1 allele (16)	major allele (17)	+1 allele (18)
MEAN%	16.11%	100%	0.44%
STDEV%	1.94%	0%	0.28%
YRI-NA18853	16.18%	100%	18.26%

Figure S2: Examples of an allele duplication and a somatic mutation deduced from read-depth observations.

a) Read-depth of the two DYS19 alleles in sample bhu-1000 (in red), compared to same-size alleles in other samples (blue) and their corresponding stutters (-1 and +1). The sample has two alleles of approximately equal read-depth, consistent with an allele duplication.

b) Read-depth of the two DYS458 alleles in sample YRI-NA18853 (red), compared to same-size alleles in other samples (blue) and their corresponding stutters (-1 and +1). As well as the major allele, the sample has a +1 allele with read-depth much greater than the stutter observed in other samples, consistent with a somatic mutation. Note that stutter at the -1 position is high compared to the +1 position, so we do not call somatic mutant alleles at the -1 position.

Refer to Table S3 for information about the relative read-depth ratio approach to distinguishing allele duplications from somatic mutations.

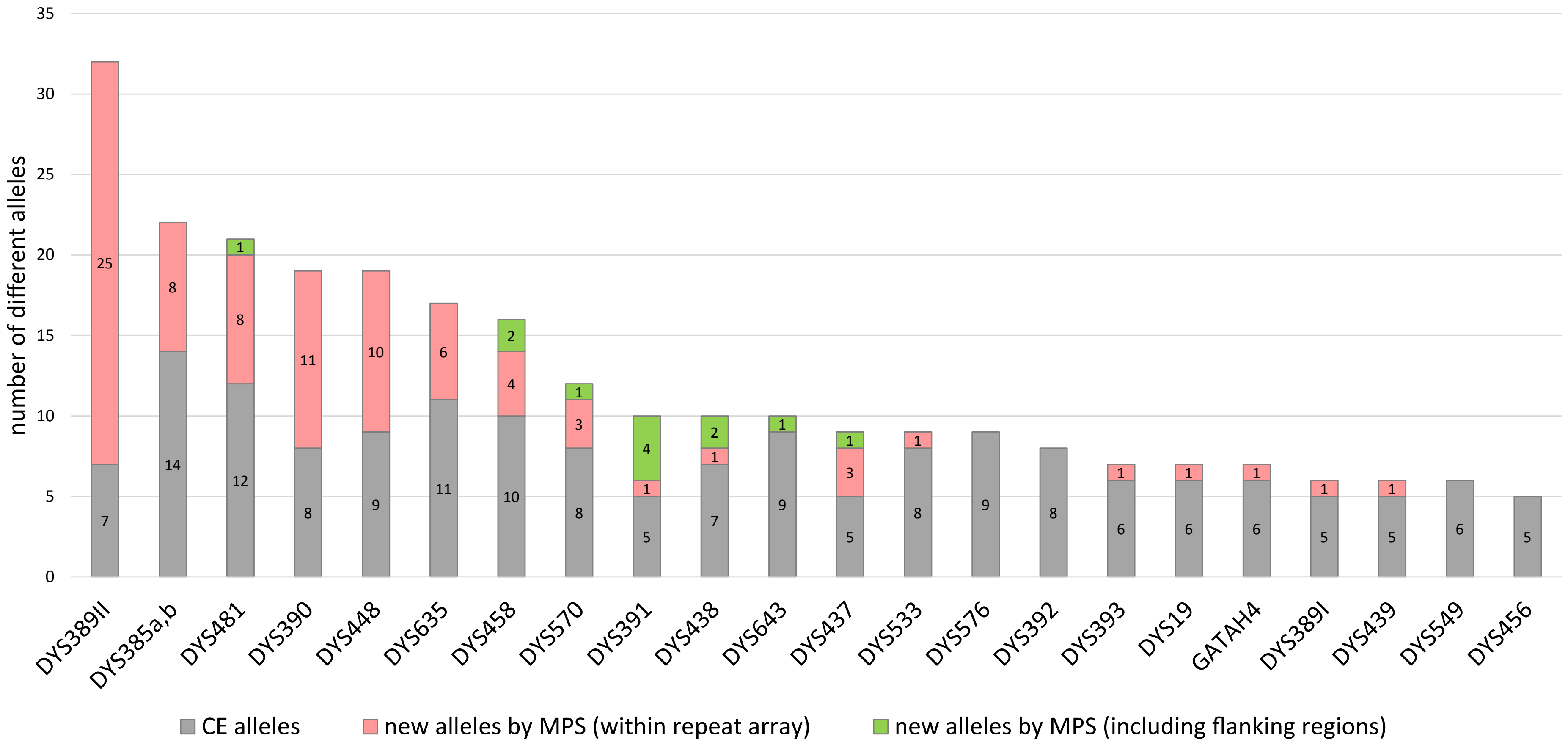

Figure S3. Increase in number of different alleles for each Y-STR using sequence information from MPS analysis.

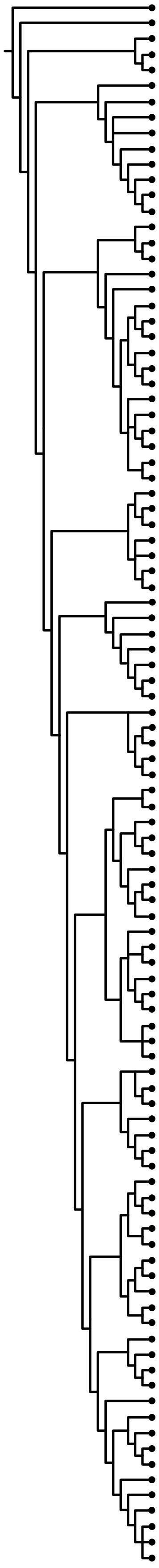

Haplogroup	Sample	DYS19	DYS385a	DYS385b	DYS389I	DYS389II	DYS390	DYS391	DYS392	DYS393	DYS437	DYS438	DYS439	DYS448	DYS456	DYS458	DYS481	DYS533	DYS549	DYS570	DYS576	DYS635	DYS643	GATA-H4
A0	bkl-46	14	15	16	13	30	20	12	14	14	16	14	13	19	15	17	25	12	12	18	18	17	7	11
A1	eng-GB1778	16	15	18	12	29	22	10	11	14	14	8	11	21	15	17	21	12	12	18	16	21	12	11
A3	man-231	15	11	12	14	31	21	10	11	13	16	11	11	23	14	17	25	10	10	16	17	19	9	12
A2	bak-41	12	17	17	13	29	22	10	13	10	16	10	11	18	14	17	25	11	11	16	18	20	12	8
A2	kun-m82	12	15	17	13	28	21	10	13	11	16	11	11	18	15	19	27	10	13	14	17	21	11	8
B2a	bak-25	15	11	12	13	31	23	11	11	13	14	10	13	21	14	15	24	11	11	16	19	17	13	12
B2b	mbu-29	16	11	12	13	30	23	8	11	13	14	11	12	21	14	18	25	15	11	17	18	18	10	12
B2b	mbu-33	14	11	17	13	29	24	10	11	14	14	10	12	20	17	15	24	12	11	20	17	18	11	10
B2b	mbu-5	15	12	9	15	30	23	9	11	13	15	10	12	22	15	13	24	12	13	16	16	18	10	13
B2b	bak-33	15	12	14	14	29	22	8	11	13	15	10	10	23	16	18	26	11	13	16	16	25	11	12
B2b	mbu-13	12	10	10	13	30	26	12	11	14	14	10	12	20.4	15	17	24	12	13	15	16	21	13	11
B2b	mbe-237P	17	9	9	12	28	23	11	--	14	15	10	12	20	17	15	27	11	12	16	14	19	10	12
B2b	bak-35	15	10	12	13	29	25	10	11	13	14	12	13	13	16	17	26	11	12	21	18	19	8	11
B2b	bak-55	16	10	10	11	28	24	9	11	12	14	11	11	19	16	18	27	9	10	20	19	21	9	12
D	bhu-1150	15	11	13	14	30	24	11	7	11	14	11	12	21	16	17	26	12	13	17	19	21	11	13
D1	bhu-0957	15	16	18	12	28	24	11	10	12	14	10	11	19	15	19	28	11	11	21	18	20	10	11
D2	JPT-NA18940	15	15	17	13	30	25	11	11	13	14	10	12	17	16	15	26	12	11	19	19	21	14	12
E2	YRI-NA19175	14	13	18	13	31	24	10	12	13	14	11	11	18	16	16	26	11	13	17	16	21	10	11
E1a	YRI-NA18856	16	14	17	12	29	22	10	11	12	17	10	13	19,20	16	16	27	11	12	17	14	21	12	11
E1b	spa-20	14	12	15	13	30	23	9	11	13	14	10	12	21	15	15	19	12	12	19	17	23	12	12
E1b	LWK-NA19334	13	16	17	14	31	24	10	11	14	14	10	11	20	15	17	25	12	12	21	17	21	12	12
E1b	bav-13	13	16	18	12	30	25	10	11	13	14	10	12	20	15	16	26	10	11	20	20	21	11	9
E1b	tur-4	13	17	18	12	30	24	10	11	13	14	10	10	20	15	14	23	12	12	21	18	21	13	11
E1b	pal-4929	14	16	17	13	29	24	10	11	13	14	10	13	20	16	15	21	12	13	18	16	20	12	12
E1b	TSI-NA20510	13	16	18	12	29	24	10	11	13	14	10	12	19	17	15	22	12	12	17	18	21	12	11
E1b	pal-5225	15	15	16	13	30	21	11	11	14	14	11	13	21	15	18	23	10	11	20	16	22	15	11
E1b	YRI-NA19098	15	15	16	13	30	21	10	11	14	14	11	12	20	17	16	26	12	11	19	16	23	13	11
E1b	YRI-NA18504	17	17	18	13	30	21	10	11	14	14	12	11	21	16	17	29	11	10	18	17	21	14	12
E1b	YRI-NA18507	16	17	18	13	31	21	10	11	14	13	11	13	21	15	15	25	11	11	16	18	21	13	11
E1b	ngo-98	15	15	20	13	30	21	10	11	14	14	11	13	21	16	17	27	12	11	20	15	21	12	12
E1b	YRI-NA18853	15	16	17	13	34	21	11	11	13	14	12	13	21	15	17,18	26	11	13	18	15	22	14	12
C2	bhu-1000	16,17	12	12	13	30	24	9	11	13	14	10	11	20	15	18	27	11	12	16	19	23	10	10
C2	CHB-NA18612	16	11	18	13	30	23	10	11	15	14	10	10	19	15	16	24	11	13	16	15	20	9	12
C2	CHB-NA18620	15	11	19	13	30	23	11	11	14	15	10	12	21	15	16	24	12	13	17	18	21	9	11
C1b	nep-0273	14	14	15	14	31	24	10	11	14	14	10	13	19	14	18	27	11	12	16	19	20	11	12
C1a	aus-m119	14	13	15	13	30	19	11	11	13	14	11	11	23	14	17	25	10	11	18	17	23	10	12
C1a	JPT-NA18974	13	13	13	13	29	24	10	11	14	14	10	12	18	15	18	27	12	12	20	20	21	11	12
C1a	nep-0172	14	14	14	13	30	24	9	11	15	14	10	11	18	14	14	28	11	12	19	14	19	9	10
G1	bav-55	14	14	14	12	30	24	10	13	13	16	10	12	21	15	18	22	10	13	17	15	21	11	10
G2a	tur-1	15	15	18	11	27	22	10	10	14	16	10	12	21	15	17	21	10	13	18	15	21	11	11
G2a	bas-19	15	13	15	12	30	21	10	11	15	16	10	11	22	15	16	21	10	12	17	16	21	12	11
G2a	pal-4919	15	14	14	12	29	23	10	11	14	16	10	12	21	15	18	21	10	13	17	18	22	11	12
G2a	hun-47	15	14	15	12	30	22	10	11	14	16	10	11	21	15	16	21	9	12	18	15	20	11	12
G2a	hun-29	15	13	14	12	29	22	11	11	14	16	10	14	20	15	17	21	9	12	17	17	21	12	10
G2a	pal-4922	13	13	14	12	29	22	10	11	14	16	10	12	22	15	16	21	9	13	18	16	20	11	11
H*	nep-0186	15	15	15	14	32	22	10	11	14	16	10	11	19	15	19	21	12	11	19	19	21	13	12
H1	bhu-1151	14	13	17	14	30	22	10	11	13	15	11	12	18	15	19	20	10	11	18	17	24	10	11
H1	nep-0809	15	14	16	14	30	21	10	11	13	15	11	12	20	14	16	23	11	11	18	18	21	11	11
H1	nep-0902	15	13	14	13	29	22	10	11	13	14	10	13	19	16	16	24	11	12	16	15	21	10	12
H1	nep-0171	15	17	17	14	30	22	11	11	12	14	9	11	19	15	16	22	12	12	16	18	20	9	12
J1	tur-16	14	14	16	13	30	23	10	11	12	14	11	12	20	14	19.2	26	11	12	19	15	20	9	12
J1	TSI-NA20543	14	14	19	13	30	22	10	11	12	14	10	12	20	15	17.2	25	11	13	18	16	20	9	11
J2b	pal-5232	15	16	18	12	30	24	11	11	12	14	9	12	19	13	16	22	12	13	21	18	21	9	11
J2b	TSI-NA20588	14	14	17	12	29	26	10	11	13	15	10	11	19	14	17	21	11	12	15	18	22	8	10
J2b	gre-2	14	14	17	12	28	24	10	11	12	16	9	14	19	13	17	23	12	13	17	16	21	9	11
J2a	tur-13	15	13	16	13	29	24	10	11	12	15	10	11	19	15	20	22	12	15	18	19	21	9	11
J2a	tur-7	14	16	17	13	30	23	10	11	12	15	9	11	19	14	16	22	11	12	16	17	22	10	12
J2a	TSI-NA20805	15	10	18	13	29	23	9	11	12	14	9	10	21	16	14	22	12	12	19	19	22	9	11
J2a	gre-77	14	13	16	14	32	23	10	11	12	14	9	11	20	16	15	25	11	11	17	18	22,23	10	11
I2	hun-5	16	13	14	12	29	21	10	11	12	15	10	11	20	15	18	26	12	11	16	17	24	12	11
I2	ire-0114	16	13	17	12	28	24	11	11	13	15	10	11	21	14	18	24	11	12	17	16	21	13	10
I2	CEU-NA12003	15	14	15	14	32	23	10	12	14	14	10	11	20	13	14	27	12	12	19	17	21	12	10
I2	gre-4	16	12	12	14	29	24	10	11	13	14	10	12	21	16	16	22	12	10	20	15	22	12	12
I2	ire-94	16	14	16	13	30	24	10	11	13	15	10	12	20	17	16	24	12	11	19	16	21	12	12
I2	ser-21	16	14	15	13	30	24	11	11	13	15	10	13	19	15	18	30	13	11	19	19	23	10	11
I1	CEU-NA11992	14	14	14	13	30	22	10	11	13	16	10	12	20	13	15	25	11	11	20	16	21	12	12
I1	CEU-NA11829	14	14	15	12	28	23	10	11	13	16	10	11	20	14	17	25	11	13	19	16	21	11	12
I1	CEU-NA06994	15	13	14	12	28	22	10	11	13	16	10	11	20	14	16	25	11	10	18	16	22	12	11
L*	tur-9	15	13	16	13	30	23	10	13	11	15	10	12	19	15	15	24	12	12	16	17	22	11	10
L1	nep-0387	14	13	17	12	28	22	10	16	11	14	11	12	19	16	15	23	12	13	16	16	24	10	12
L1	bhu-1611	15	17	9	13	29	22	10	14	12	16	11	11	19	15	18	25	11	11	16	19	21	11	10
T1a	bhu-1892	15	16	18	13	29	23	9	13	13	14	9	13	19	15	15	23	11	12	18	16	22	10	11
T1a	TSI-NA20527	14	16	16	14	31	23	10	14	13	14	9	12	19	16	17	22	12	13	17	16	21	10	11
T1a	pal-5366	13	14	15	13	30	23	11	14	13	14	9	13	19	15	15	27	11	12	18	17	21	11	11
T1a	gre-12	13	13	14,15	14	30	22	10	13	13	15	9	11	19	16	16	24	12	13	17	18	21	12	12
O1a	CHB-NA18632	15	13	14	12	28	22	10	14	13	14	10	11	18	14	15	26	11	14	18	16	22	10	12
O1b	CHB-NA18561	16	10	19	14	29	23	10	13	13	14	13	12	18	15	17	21	11	12	18	21	21	13	11
O1b	CHB-NA18637	15	13	17	13	29	24	11	13	14	14	10	11	18	15	15	25	11	13	18	17	22	11	12
O3	CHB-NA18621	15	13	22	13	29	24	12	13	13	15	10	12	20	15	16	23	11	13	19	17	20	12	11
O3	CHB-NA18622	14	13	19	12	27	24	11	14	12	15	11	11	20	15	18	23	11	12	20	19	20	12	11
N	CHB-NA18558	14	11	12	13	30	22	10	14	13	14	10	10	19	16	17	21	12	12	21	16	23	11	12
N	CHB-NA18608	14	11	12	12	28	22	10	14	13	14	10	10	19	15	16,17	21	12	12	17	16	21	9	12
N	bhu-1142	14	12	12	13	31	22	11	15	13	14	11	11	19	15	15	22	12	14	17	17	24	10	12
N	gre-192	14	12	13	13	29	23	10	14	13	14	10	10	17	15	16	21	13	12	18	16	25	10	12
N	saa-15	14	11	13	14	30	24	11	14	14	14	10	10	18	14	17	21	11	11	17	18	21	12	12
Q1b	eng-hgQ-2	13	14	17	13	29	22	10	15	13	14	11	12	19	15	17	24	12	12	17	17	22	12	10
Q1a	bhu-1564	13	15	21	14,15	30,31	24	9	14	13	14	12	11	19	16	16	25	12	12	19	19	22	10	10
Q1a	eng-hgQ-1	13	13	17	12	29	23	10	12	13	14	11	12	19	17	16	20	11	12	17	20	22	10	11
Q1a	MXL-NA19664	14	14	17	13	31	24	10	14	13	15	11	11	18	14	16	24	14	12	18	16	21.3	10	11
R2	bhu-1157	14	13	19	14	30	23	10	10	14	16	11	11	20	17	18	22	11	14	20	15	26	11	12
R1a	ork-026m	15	14	14	14	32	26	11	13	13	14	11	10	19	17	16	23	12	12	18	17	23	11	11
R1a	nor-16	17	11	14	13	30	24	10	11	13	14	11	10	19	16	15	24	11	13	17	18	23	10	11,13
R1a	ser-19	16	11	14	13	30	25	11	11	13	14	11	10	20	15	15	23	12	12	19	18	23	11	11
R1a	ork-036m	15	11	13	13	30	25	11	11	13	14	11	10	20	16	15	23	12	12	20	17	23	10	13
R1b	bhu-0984	15	11	13	14	30	24	10	13	13	14	12	11	21	15	15	21	11	12	21	18	24	13	12
R1b	gre-10	14	12	14	14	30	23	11	13	12	14	12	14	18	16	16	22	12	13	16	17.1	23	11	12
R1b	den-190	14	11	14	13	29	23	11	13	13	15	12	12	19	15	18	22	12	13	18	19	23	10	12
R1b	ork-007	16	11	15	14	30	24	11	13	13	16	11,12	14	19	16	18	23	12	11	17	18	23	9	11
R1b	ire-0068	14	11	13	13	29	26	12	14	13	14	11	12	18	17	18	26	13	12	17	17	23	10	12
R1b	CEU-NA12716	14	11	14	12	28	24	11	13	13	15	12	12	19	16	17	23	12	12	17	19	23	10	11

Figure S4: Length-based Y-STR alleles from MPS data in the 100 samples, in their phylogenetic context.

To the left is the schematic phylogeny relating the MSY haplogroups of the 100 samples in this study, with haplogroup and sample names (as in Table S1) given at the tips. To the right are shown the Y-STR allele length-based designations, with Y-STR names given at the top. For each Y-STR, cells containing allele names are heat-map coloured from blue (shortest) to red (longest). The four discordances listed in Table S4 are underlined.
